# Silymarin Attenuates Arthritis and Myositis in a Murine Model of Acute Infection by Chikungunya and Mayaro Viruses

**DOI:** 10.1021/acsinfecdis.5c00901

**Published:** 2026-01-23

**Authors:** Rafaela Lameira Souza Lima, Ariane Coelho Ferraz, Marília Bueno da Silva Menegatto, Oluwashola Samuel Ola-Olu, Pedro Henrique Guimarães, Giovana Mesquita Oliveira de Castro Domingos, Allen Rene Ruiz Hernández, Maria Eduarda Diniz Starling, Pedro Alves Machado-Junior, Frank Silva Bezerra, José Carlos de Magalhães, Wanderson Geraldo de Lima, Cintia Lopes de Brito Magalhães

**Affiliations:** † Postgraduate Program in Biological Sciences, Research Center in Biological Sciences, 28115 Federal University of Ouro Preto, Ouro Preto, Minas Gerais 35400-000, Brazil; ‡ Postgraduate Program in Biotechnology, Research Center in Biological Sciences, 28115 Federal University of Ouro Preto, Ouro Preto, Minas Gerais 35400-000, Brazil; § Medical Course, 28115 Federal University of Ouro Preto, Ouro Preto, Minas Gerais 35400-000, Brazil; ∥ Postgraduate Program in Biotechnology, Federal University of São João Del-Rei, São João Del-Rei, Minas Gerais 36307-352, Brazil

**Keywords:** *Alphavirus chikungunya*, *Alphavirus mayaro*, arthritis, myositis, silymarin

## Abstract

The alphaviruses chikungunya (CHIKV) and Mayaro (MAYV) are responsible for acute febrile illnesses often accompanied by severe and persistent joint and muscle pain. Due to the lack of specific treatment, research into antivirals against these emerging viruses is seen as an urgent need. Previous studies demonstrated that silymarin exhibits potent antiviral activity against CHIKV and MAYV. Then, given the promising antiviral profile of silymarin, and the prominent joint and muscle pain caused by these viruses, we evaluated whether silymarin could reverse these damages in a murine model of alphavirus-induced arthritis and myositis. BALB/c mice were infected with CHIKV or MAYV in the right hind paw pad, and treated groups received silymarin orally (200 mg/kg/day). Clinical observation revealed reduced paw edema in silymarin-treated animals. At 7 and 12 days postinfection (dpi), animals were euthanized and various tissues collected. In infected and treated animals, a greater than 90% reduction in CHIKV viral load was observed in the spleen (7 and 12 dpi), paw (7 dpi), soleus muscle, and liver (12 dpi). Similarly, for MAYV, a greater than 90% reduction in viral load was detected in the spleen (7 and 12 dpi), liver, quadriceps, soleus muscle (7 dpi), and paw (12 dpi). Histological analysis revealed reduced inflammatory infiltrates in the liver, paw, and muscles as well as a decrease in both the number and area of lymphoid nodules in the spleen (12 dpi). Furthermore, silymarin treatment reduced TNF-α levels by at least 2-fold in the paw (7 and 12 dpi) and quadriceps (12 dpi). These findings suggest that silymarin not only limits viral replication in key target tissues, including the spleen, liver, muscle, and paw, but also mitigates inflammation by reducing paw edema, inflammatory infiltrates in hepatic, musculoskeletal, and paw tissues, the number and area of lymphoid nodules in the spleen, and TNF-α levels in the quadriceps muscle and paw, thereby supporting its therapeutic potential against CHIKV and MAYV infections.

## Graphical abstract

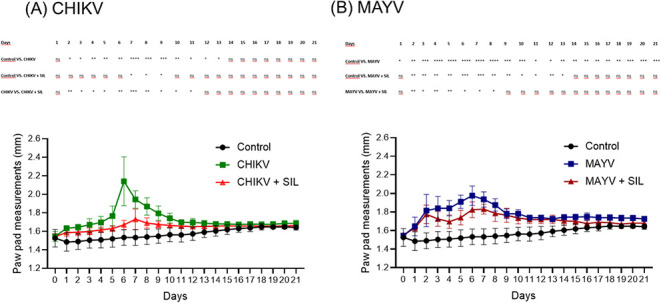

The *Alphavirus chikungunya* (CHIKV), the etiological agent of chikungunya fever (CF), is a mosquito-borne alphavirus that has recently reemerged in many parts of the world causing large-scale outbreaks.^1^ The *Alphavirus mayaro* (MAYV) is the etiological agent of Mayaro fever (MF), a neglected and endemic disease that primarily affects South American countries, where it is responsible for sporadic outbreaks.^2–4^ CHIKV and MAYV belong to the same family of viruses (*Togaviridae*) and the same genus (*Alphavirus*), causing a very similar disease. They are characterized by an acute, nonfatal, self-limiting febrile illness, characterized by headache, skin rash, ocular pain, myalgia, and disabling arthralgia.^4,5^ Infections caused by these arboviruses have gained prominence in recent years due to their increasing incidence and the persistence of symptoms beyond the acute phase, including prolonged musculoskeletal manifestations that significantly impair quality of life.^5,6^ Despite this clinical burden, no specific antiviral treatments are currently available, and management remains limited to symptomatic relief,^3,7^ underscoring the need for novel antiviral therapies.

In this context, our research group aims to elucidate some of the factors contributing to the pathogenesis of CHIKV and MAYV. We also actively search for natural substances with potential antiviral activity. In a recent study, we demonstrated that silymarin, an antioxidant phytotherapeutic agent derived from *Silybum marianum*, showed significant antiviral activity against MAYV in HepG2 cells.^2^ Subsequently, in a nonlethal mouse model, silymarin exhibited hepatoprotective, antioxidant, anti-inflammatory, and antiviral activity in MAYV-infected animals.^8^ Furthermore, other authors have already shown that silymarin has significant *in vitro* anti-CHIKV activity.^9^ However, despite these findings, its potential to modulate CHIKV- and MAYV-induced joint and muscle pathology remains unknown, largely due to the lack of appropriate *in vivo* models for arthritis and myositis induced by these arboviruses.

Silymarin is a complex of substances extracted from the plant *Silybum marianum* (*Asteraceae*). The extract from the entire plant material of *Silybum marianum* contains a mixture of flavonolignans: Silybin A, Silybin B, Isosilybin A, Isosilybin B, Silychristin, Isosilychristin, and Silydianin; in addition to the flavonoid Taxifolin.^10^ Currently, numerous studies have demonstrated the treatment of various pathologies with the use of silymarin. The increase in studies related to silymarin is believed to be due to its antioxidant and anti-inflammatory properties, as well as its modulatory role in cellular signaling pathways.^11^

Considering that joint and muscle pain are among the most prominent symptoms of CF and MF, and that silymarin has already demonstrated potent antiviral activity against CHIKV (*in vitro*) and MAYV (*in vitro* and *in vivo*), our aim was to evaluate the antiviral activity of silymarin in an animal model of arthritis and myositis induced by CHIKV and MAYV. Once these actions have been proven, silymarin could be considered a potential herbal medicine for the treatment of CHIKV and MAYV infections.

## Results

### Silymarin Reduces Paw Edema in CHIKV- and MAYV-Infected Mice

Infection of BALB/c mice with CHIKV or MAYV resulted in a nonlethal disease, and no overt clinical signs such as piloerection, prostration, weight loss, hind limb weakness, or impaired locomotion were observed during the 21-day follow-up period. In contrast, both infections induced significant paw edema. CHIKV-infected mice developed marked paw swelling, with a peak at 6 dpi, while silymarin treatment significantly attenuated edema, particularly during the acute phase of infection (Figure 1A). Similarly, MAYV infection led to sustained paw edema, also peaking at 6 dpi, and silymarin administration effectively reduced swelling during this period (Figure 1B). Overall, silymarin treatment limited virus-induced paw edema without altering the nonlethal clinical course of either infection. Two-way ANOVA revealed significant effects of time (*p* < 0.0001), treatment (*p* < 0.0001), and their interaction (*p* < 0.0001), indicating that silymarin treatment not only reduced paw edema but also modified its temporal progression during infection.

**Figure 1:**
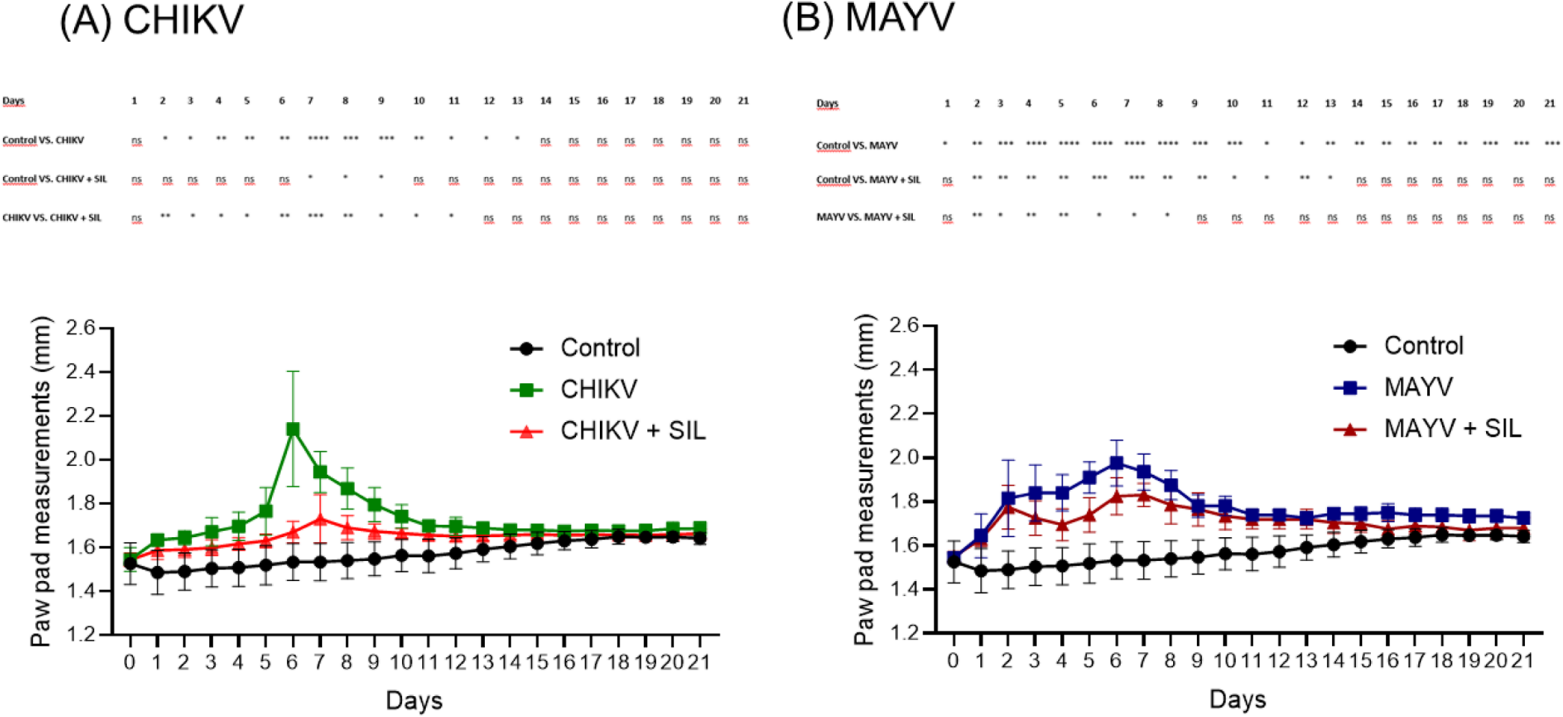
Silymarin treatment attenuates paw edema in BALB/c mice infected with CHIKV or MAYV. (A) CHIKV-infected mice. (B) MAYV-infected mice. Animals in the control group (*n* = 8) received an injection of culture medium into the right hind paw pad. Infected groups (*n* = 8) were inoculated with 10^6^ plaque-forming units (PFU) of either CHIKV or MAYV via the same route. Silymarin-treated animals received 200 mg/kg/day of silymarin by oral gavage every 12 h, starting 6 h postinfection, for a total of 21 days. Control and infected groups received vehicle solutions without silymarin. Paw thickness was monitored daily, and results are expressed as the mean ± standard deviation (SD). Statistical significance was determined using two-way ANOVA. The symbols *, **, ***, and **** indicate *p* ≤ 0.05, *p* ≤ 0.01, *p* ≤ 0.001, and *p* ≤ 0.0001, respectively.

### Silymarin Treatment Reduces Viral Load in CHIKV- and MAYV-Infected Mice

In assessing the antiviral action of silymarin, samples of liver, spleen, paws, quadriceps, EDL, tibialis anterior, and soleus muscles were used. At 7 dpi, silymarin administration significantly reduced CHIKV viral load in the spleen, corresponding to a 96.76% decrease (Figure 2B), and in the paw pad, corresponding to a 99.83% decrease (Figure 2C). However, no statistically significant differences were observed in the liver (Figure 2A) or soleus muscle (Figure 2D) at this time point. By 12 dpi, the antiviral effect of silymarin became more pronounced, with significant decreases in viral RNA levels in the liver (90.67% decrease) (Figure 2A), spleen (99.38% decrease) (Figure 2B), and soleus muscle (99.93% decrease) (Figure 2D), compared to the untreated CHIKV-infected group. In contrast, the viral load in the paw pad (Figure 2C) at 12 dpi did not differ significantly between treated and untreated animals. Notably, no viral RNA was detected in the quadriceps, tibialis anterior, or EDL muscles in any group at either 7 or 12 dpi.

**Figure 2:**
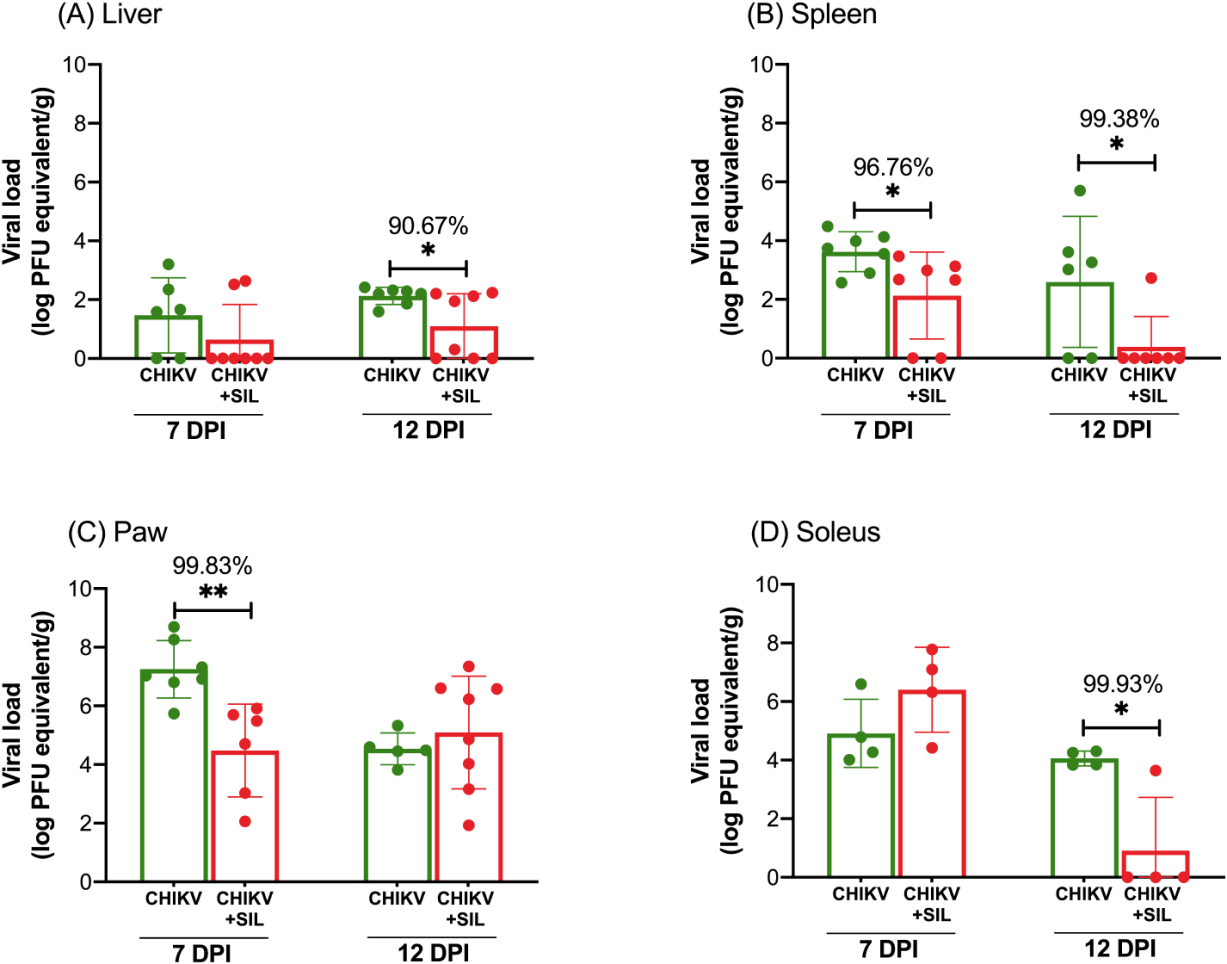
Silymarin treatment reduces CHIKV viral load in BALB/c mice. Total RNA was extracted from the liver, spleen, paw, quadriceps, tibialis anterior, soleus muscle, and extensor digitorum longus (EDL) muscles. Viral load was quantified by RT-qPCR using a standard curve generated from CHIKV RNA of a known concentration. Mice were infected with CHIKV via injection into the right hind paw and treated with silymarin (200 mg/kg/day) via oral gavage every 12 h, starting 6 h postinfection, for 7 or 12 days. A vehicle solution (without silymarin) was administered to the infected control groups. No CHIKV RNA was detected in the tibialis anterior, quadriceps, or EDL muscles. Data are expressed as mean ± SD. Statistical significance was determined using Student’s *t* test, with symbols *, **, and *** indicating *p* ≤ 0.05, *p* ≤ 0.01, and *p* ≤ 0.001, respectively.

Regarding MAYV infection, silymarin treatment led to a significant reduction in viral load in the liver (89.04% decrease) (Figure 3A), spleen (97.55% decrease) (Figure 3B), quadriceps (96.20% decrease) (Figure 3C), and soleus muscles (99.97% decrease) (Figure 3E) at 7 dpi. At 12 dpi, the reduction in viral load persisted in the spleen (91.87% decrease) (Figure 3B) and paw (99.98% decrease) (Figure 3D). However, viral RNA levels in the liver, quadriceps, soleus, tibialis anterior, and EDL muscles did not differ significantly between treated and untreated animals at this later time point. Across all groups and time points, the viral load in the tibialis anterior (Figure 3F) and EDL (Figure 3G) muscles remained statistically unchanged.

**Figure 3:**
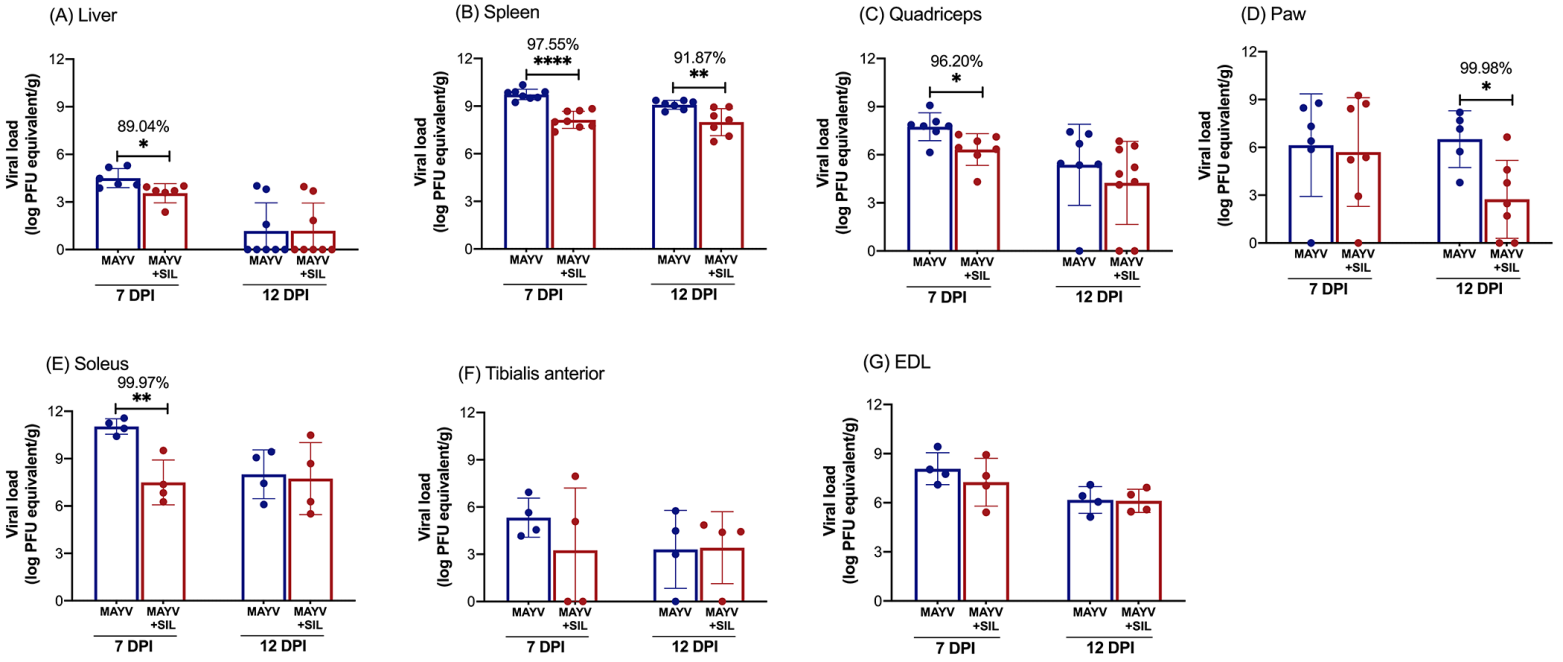
Silymarin treatment reduces MAYV viral load in BALB/c mice. Total RNA was extracted from the liver, spleen, paw, quadriceps, tibialis anterior, soleus, and extensor digitorum longus (EDL) muscles. Viral load was quantified by RT-qPCR using a standard curve generated from MAYV RNA of a known concentration. Mice were infected with MAYV via injection into the right hind paw and treated with silymarin (200 mg/kg/day) via oral gavage every 12 h, starting 6 h postinfection, for 7 or 12 days. A vehicle solution (without silymarin) was administered to the infected control groups. Data are expressed as mean ± SD. Statistical significance was determined using Student’s *t* test, with symbols *, **, and *** indicating *p* ≤ 0.05, *p* ≤ 0.01, and *p* ≤ 0.001, respectively.

### Silymarin Attenuates Liver and Spleen Injuries Induced by CHIKV and MAYV

Infection with CHIKV and MAYV induced marked hepatic damage in mice, as demonstrated by histological and morphometric analyses. In the CHIKV group, liver pathology at 7 days postinfection (dpi) was characterized by hemorrhage, necrosis, and inflammatory infiltrates within the hepatic parenchyma (Figure 4A). At 12 dpi, inflammatory infiltrates persisted and were accompanied by pronounced hyperemia. In contrast, silymarin treatment (CHIKV + SIL) reduced hemorrhage and inflammatory infiltrates; at 7 dpi, only mild degenerative changes were observed, which were no longer evident at 12 dpi, indicating tissue recovery. Signs of hyperemia were observed in both CHIKV and CHIKV + SIL groups at both time points. Morphometric analysis confirmed a significant increase in inflammatory cell counts in CHIKV-infected animals at 7 and 12 dpi compared with control mice, while silymarin treatment reduced inflammatory cell numbers to levels not significantly different from controls (Figure 4B; Supplementary Table S1).

**Figure 4:**
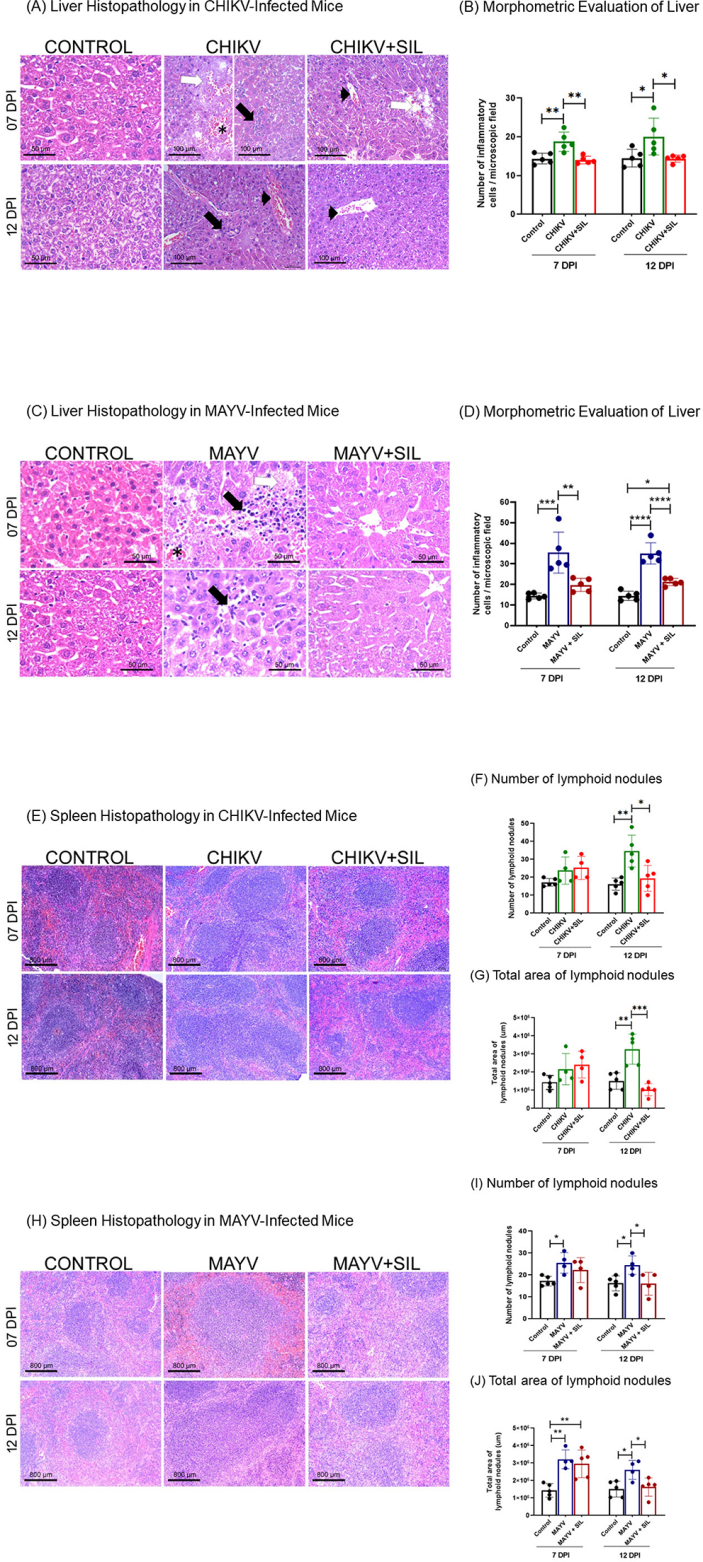
Silymarin improves liver and spleen pathology in BALB/c mice infected with CHIKV or MAYV. Photomicrographs of hematoxylin and eosin-stained liver (A, C) and spleen (E, H) sections. (A) Liver sections from control animals displayed normal architecture. CHIKV-infected animals showed necrosis (white arrow), hemorrhage (asterisk), inflammatory cell infiltration (black arrow), and hyperemia (arrowhead). CHIKV-infected animals treated with silymarin exhibited mild degenerative changes (white arrow) and hyperemia (arrowhead), with an overall improvement in tissue integrity. (C) Liver sections from MAYV-infected animals demonstrated necrosis (white arrow), hemorrhage (asterisk), and inflammatory infiltrates (black arrow), while control animals showed normal histology. Treatment with silymarin preserved liver architecture and reduced pathological features. (B, D) Morphometric analysis of inflammatory cell counts in liver sections showed a significant reduction in inflammatory infiltrates in both CHIKV- and MAYV-infected mice treated with silymarin. (E) In CHIKV-infected animals, spleen histology at 12 dpi revealed an increased number and area of lymphoid nodules, which were markedly reduced following silymarin treatment. (H) Similarly, MAYV-infected animals exhibited enlarged and more numerous lymphoid nodules compared to controls; these alterations were attenuated by silymarin treatment at 12 dpi. (F, I) Quantification of lymphoid nodule numbers and (G, J) total nodule area support the histopathological findings. Data are presented as mean ± SD. Statistical significance was determined using one-way ANOVA, with symbols *, **, ***, and **** indicating *p* ≤ 0.05, *p* ≤ 0.01, *p* ≤ 0.001, and *p* ≤ 0.0001, respectively. Spleen: original magnification 100× and scale bar = 800 μm. Liver (control): original magnification 400× and scale bar = 50 μm. Liver (CHIKV and CHIKV + SIL): original magnification 200 × and scale bar = 100 μm. Liver (MAYV and MAYV + SIL): original magnification 400× and scale bar = 50 μm.

Similarly, MAYV-infected animals exhibited severe hepatic alterations at 7 days postinfection, including inflammatory infiltrates, necrosis, and hemorrhage, with persistent inflammatory infiltration remaining the predominant feature at 12 days postinfection (Figure 4C). Silymarin treatment (MAYV + SIL) attenuated these histopathological alterations, consistent with a hepatoprotective and anti-inflammatory effect. Morphometric analysis confirmed a significant reduction in inflammatory cell counts in the liver parenchyma of MAYV-infected animals treated with silymarin (Figure 4D; Supplementary Table S1).

Histopathological and morphometric evaluation of the spleen revealed an increased number and total area of lymphoid nodules in CHIKV-infected animals at 12 dpi and in MAYV-infected animals at both 7 and 12 dpi, compared to uninfected controls (Figure 4E–J). Silymarin treatment exhibited promising effects, significantly reducing the number and area of lymphoid nodules in CHIKV- and MAYV-infected animals at 12 dpi, compared to untreated infected animals. Notably, no significant differences were observed between silymarin-treated and control groups at this point in time, supporting its immunomodulatory and anti-inflammatory potential (Figure 4E–J).

### Silymarin Mitigates Alphavirus-Induced Myositis by Reducing Inflammatory Infiltrates in Skeletal Muscles of CHIKV- and MAYV-Infected Animals

Histological analysis of the quadriceps, soleus, tibialis anterior, and EDL muscles revealed pathological changes consistent with CHIKV (Figure 5A,E,I,M) and MAYV (Figure 5C,G,K, and O) infection, characterized by the presence of focal inflammatory infiltrates within the muscle tissue, indicative of virus-induced myositis. Morphometric evaluation further confirmed a significant increase in the number of inflammatory cells in CHIKV (Figure 5B,F,J,N) and MAYV (Figure 5D,H,L,P) infected muscles compared to noninfected controls. Silymarin administration exerted a clear anti-inflammatory effect, as evidenced by a reduction in inflammatory cell infiltration in all treated muscles, thereby mitigating the severity of myositis.

**Figure 5:**
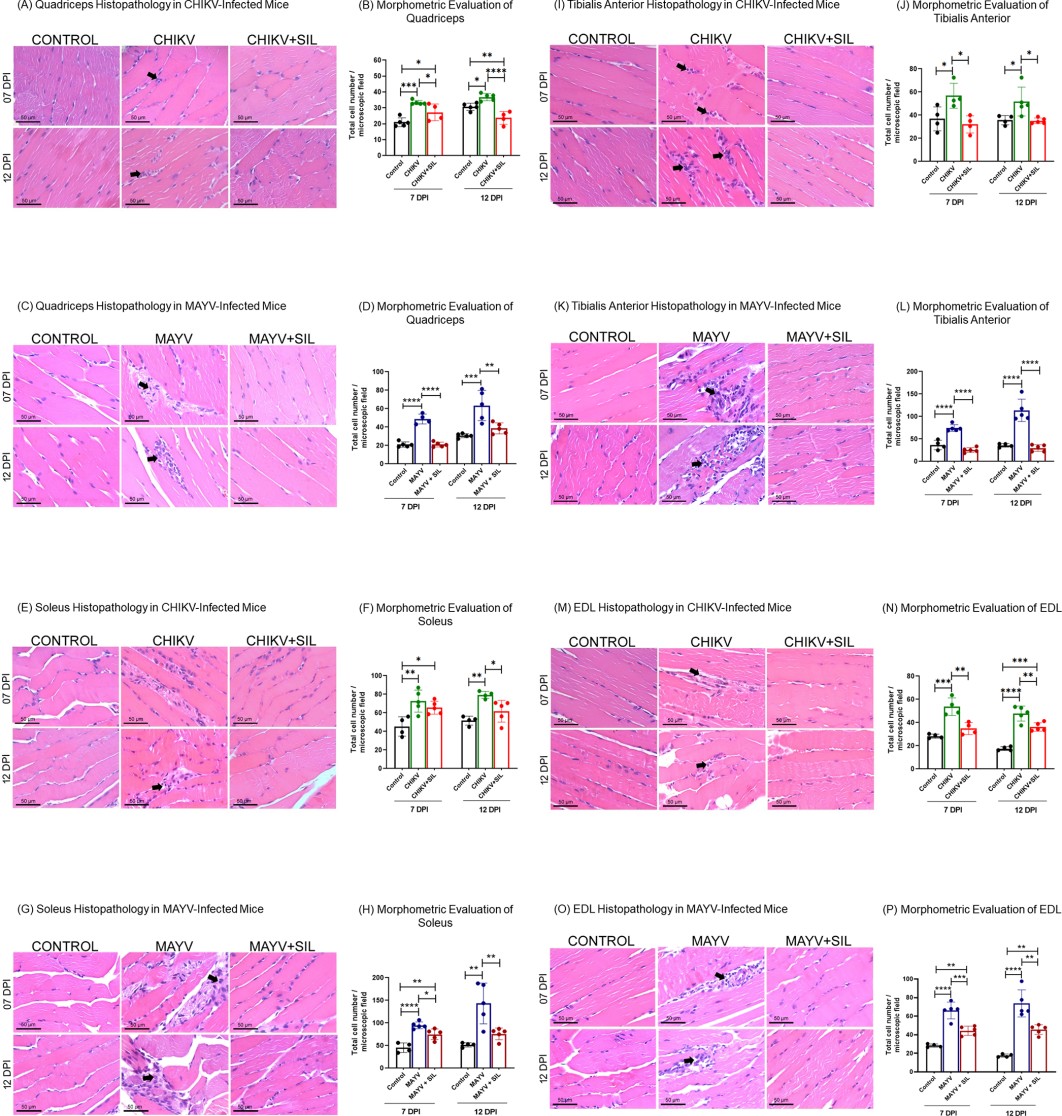
Silymarin attenuates myositis by reducing inflammatory cell infiltration in skeletal muscles of CHIKV- and MAYV-infected BALB/c mice. Photomicrographs of histological sections of the quadriceps (A, C), soleus (E, G), tibialis anterior (I, K), and EDL (M, O) muscles stained with hematoxylin and eosin. Black arrows indicate foci of inflammatory cell infiltration characteristic of virus-induced myositis. Panels B,D,F,H,J,L,N,P present morphometric quantification of total inflammatory cells in the corresponding muscle sections. Silymarin treatment significantly reduced inflammatory infiltrates in most muscle groups evaluated for both CHIKV and MAYV infections. The only exception was the soleus muscle of the CHIKV group at 7 days postinfection, where no statistically significant reduction was observed in treated animals compared to infected controls (F). Data are presented as mean ± SD. Statistical significance was determined using one-way ANOVA, with symbols *, **, ***, and **** indicating *p* ≤ 0.05, *p* ≤ 0.01, *p* ≤ 0.001, and *p* ≤ 0.0001, respectively. Original magnification 400× and scale bar = 50 μm.

### Therapeutic Potential of Silymarin in Modulating Inflammatory Responses in Alphavirus Arthritis

Histopathological and morphometric analyses were conducted on muscle, bone marrow, and articulation tissues from the hind paws of infected animals at 7 and 12 dpi. In Figure 6 (A–C), an increase in inflammatory cells can be observed in the muscle and bone marrow in the paws of animals infected with CHIKV on days 7 and 12 postinfection, and treatment with silymarin (CHIKV + SIL) was able to significantly reduce inflammation in infected animals on different days. Similarly, MAYV infection led to increased inflammation in the muscles and bone marrow of the paws of animals on different days, and silymarin significantly reduced inflammation in all infected groups (Figure 6D–F).

**Figure 6:**
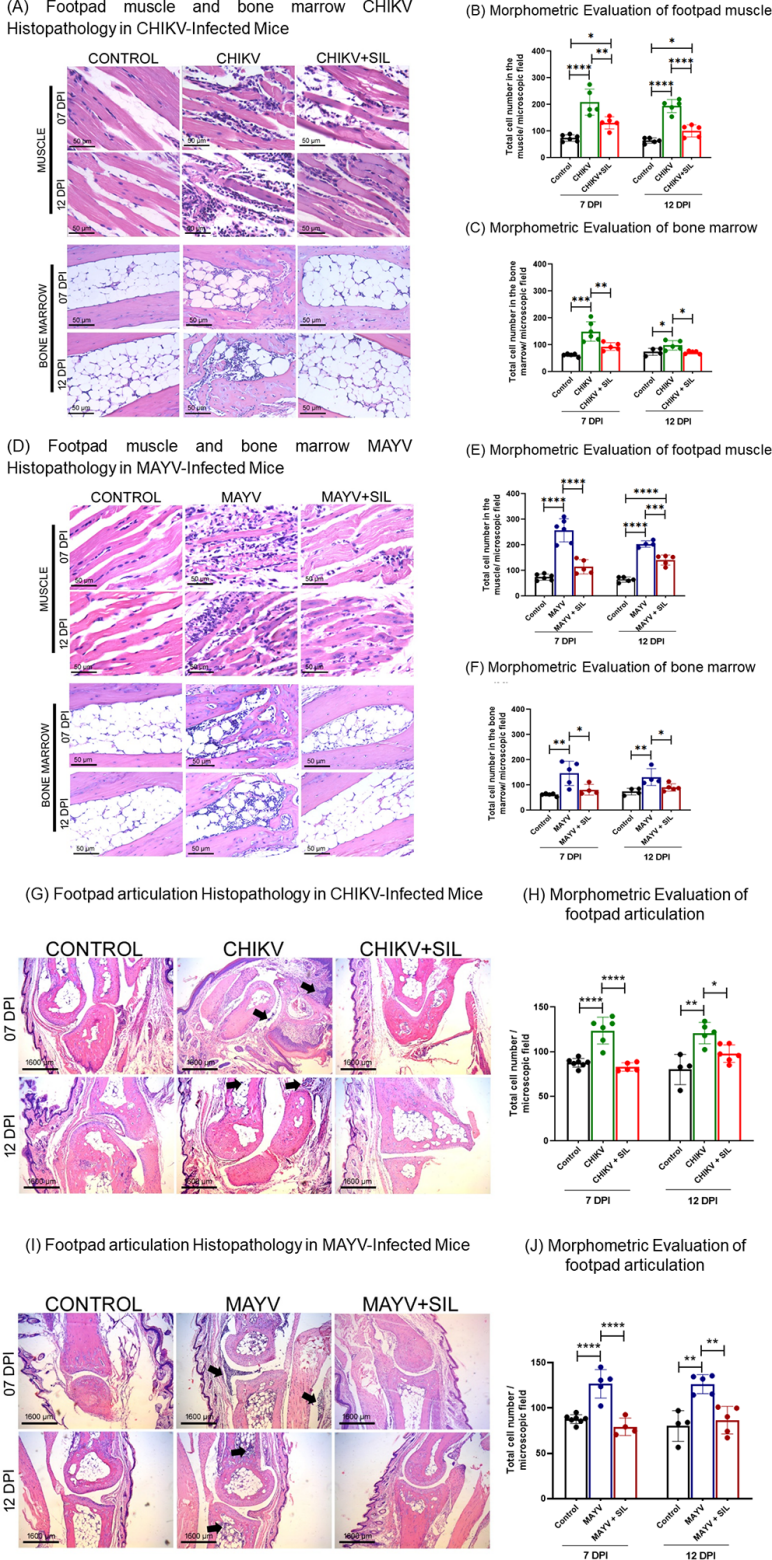
Anti-inflammatory effects of silymarin in CHIKV- and MAYV-induced arthritis. Photomicrographs of hematoxylin and eosin-stained sections of paw muscle and bone marrow (A, D) and paw joint (G, I). (A, D) Control animals exhibited normal histological architecture in muscle and bone marrow from the hind paw. In contrast, CHIKV- and MAYV-infected animals showed marked inflammatory cell infiltration and increased cellularity in both tissues, which were significantly reduced following silymarin treatment, as confirmed by morphometric analysis (B,C and E,F). (G) Joint sections from CHIKV-infected animals displayed pronounced inflammatory infiltrates in the synovial membranes and adjacent tissues, including the epidermis, bone marrow, and muscles (black arrow), whereas silymarin-treated animals showed reduced inflammation (H). (I) Similar joint pathology was observed in MAYV-infected animals, with inflammatory infiltrates extending to adjacent tissues, such as bone marrow and muscle (black arrow); treatment with silymarin markedly attenuated these alterations, as quantified in panel (J). Data are presented as mean ± SD. Statistical significance was determined using one-way ANOVA, with symbols *, **, ***, and **** indicating *p* ≤ 0.05, *p* ≤ 0.01, *p* ≤ 0.001, and *p* ≤ 0.0001, respectively. Joints: original magnification 40× and scale bar = 1600 μm. Bone marrow and muscle: original magnification 400× and scale bar = 50 μm.

Both CHIKV and MAYV infections induced characteristic signs of arthritis, with prominent inflammatory infiltrates observed within and around the synovial membranes, particularly at 7 days postinfection (dpi) (Figure 6G,I). Inflammatory infiltrates were also detected in adjacent tissues. Pathological alterations were noted in the skeletal muscle, bone marrow, and epidermis (black arrows). Quantitative analysis confirmed a significant increase in the number of inflammatory cells in the paw joint of infected animals compared to their respective controls, highlighting a robust inflammatory response to viral infection (Figure 6H,J). Silymarin treatment markedly reduced inflammatory infiltrates across all evaluated compartments, including muscle, bone marrow, and joints, in both infection models. This attenuation of virus-induced inflammation supports the potential of silymarin as a therapeutic agent for mitigating arthritogenic outcomes associated with CHIKV and MAYV infections.

### Silymarin Reduces Proinflammatory TNF-α Expression

The elevated TNF-α production is a well-established biomarker of arthritis associated with CHIKV and MAYV infections.^12^ In order to assess the inflammatory response elicited by these viral infections and the potential anti-inflammatory effects of silymarin, TNF-α expression was quantified in the quadriceps muscles and paws using RT-qPCR. In both CHIKV- and MAYV-infected animals, a significant upregulation of TNF-α expression was detected in the quadriceps and paws compared with uninfected controls at 7 and 12 dpi (Figure 7). In the quadriceps, silymarin treatment (CHIKV + SIL and MAYV + SIL groups) led to a marked reduction in TNF-α expression at 12 dpi (Figure 7A,B), while the reduction in the paw was observed at 7 and 12 dpi (Figure 7C,D), indicating the capacity of the phytotherapeutic to attenuate alphavirus-induced arthritis.

**Figure 7:**
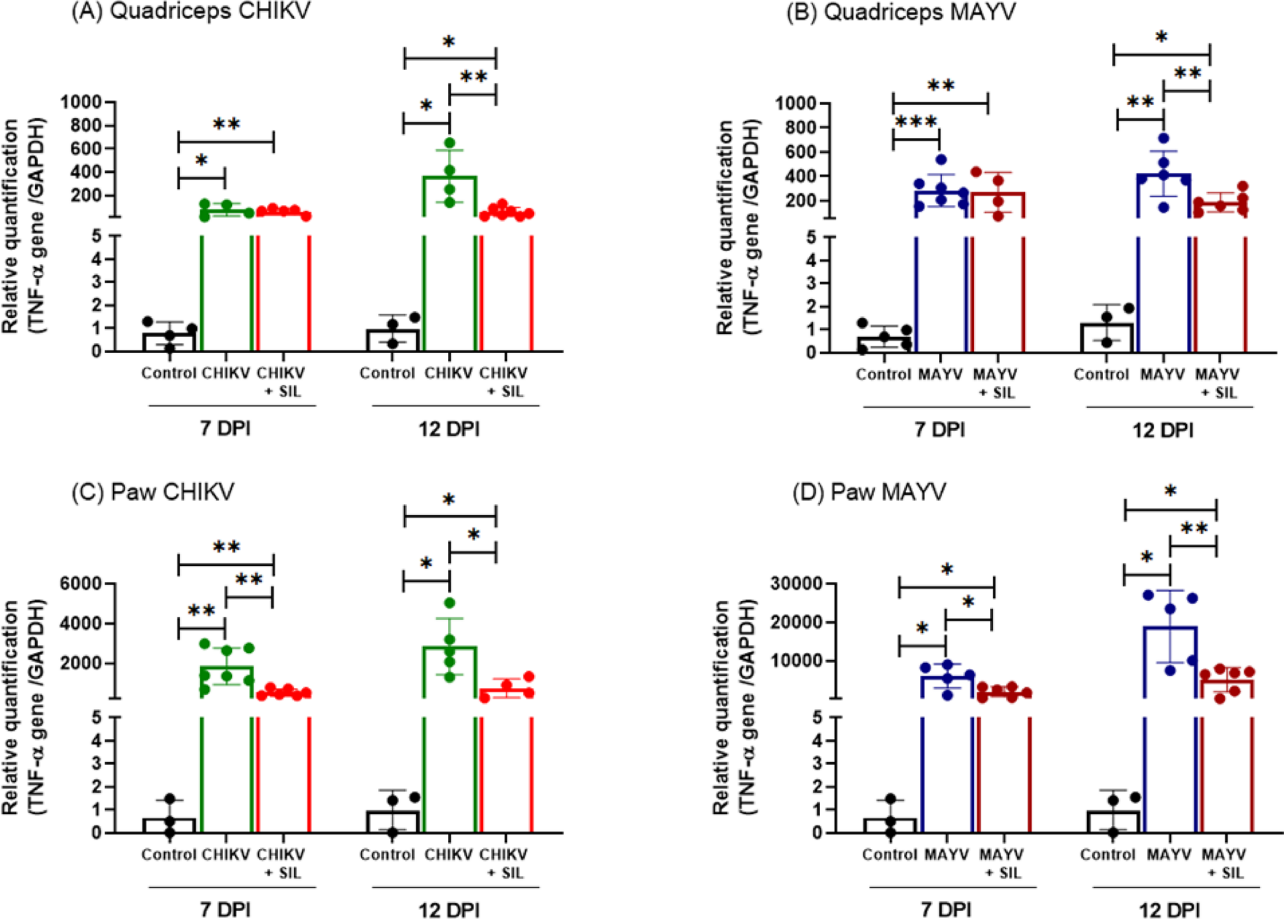
Silymarin treatment downregulates TNF-α expression in the quadriceps and paw tissues of CHIKV- and MAYV-infected mice. Total RNA was extracted from quadriceps and paw samples, and TNF-α expression levels were quantified by RT-qPCR. Silymarin administration (200 mg/kg/day) significantly reduced TNF-α mRNA expression in both tissues compared to untreated infected animals. Data are expressed as mean ± SD. Statistical significance was determined using Student’s *t* test, with symbols *, **, and *** indicating *p* ≤ 0.05, *p* ≤ 0.01, and *p* ≤ 0.001, respectively.

Importantly, TNF-α expression levels positively correlated with morphometric parameters of tissue inflammation in paw tissues at both 7 and 12 dpi, and in quadriceps tissues at 12 dpi, in CHIKV- and MAYV-infected animals. In CHIKV infection, TNF-α expression showed a moderate positive correlation with inflammatory morphometric parameters in the paw at 7 dpi (Spearman ρ = 0.5387, *p* = 0.0211) and at 12 dpi (Pearson *r* = 0.5841, *p* = 0.0138), as well as in the quadriceps at 12 dpi (Pearson *r* = 0.5703, *p* = 0.0332). In MAYV-infected animals, TNF-α expression correlated with inflammatory parameters in the paw at 7 dpi (Spearman ρ = 0.5141, *p* = 0.0437) and at 12 dpi (Pearson *r* = 0.6022, *p* = 0.0227), and showed a strong correlation in the quadriceps at 12 dpi (Spearman ρ = 0.9385, *p* < 0.0001). These findings reinforce the association between TNF-α expression and the severity of local inflammatory damage and support the anti-inflammatory effects of silymarin observed at the histological level.

## Discussion

Since infection with the alphaviruses CHIKV and MAYV can lead to joint and muscle manifestations, such as arthritis and myositis, which significantly impair the quality of life, and no specific treatment is currently available, we sought to investigate a potential antiviral candidate. Our study demonstrated that the subcutaneous inoculation of CHIKV or MAYV into the mouse paw induces clinical signs similar to those observed in human infections, including paw edema, arthritis, and myositis. Notably, paw edema is a characteristic sign of an alphavirus infection. Previous studies using C57BL/6 mice have demonstrated that CHIKV infection causes footpad swelling, with peaks between 6 and 8 dpi.^1^ Paw edema has also been previously demonstrated in MAYV-infected C57BL/6 mice.^13^ Furthermore, Mota et al. showed that MAYV infection in BALB/c mice induces persistent hypernociception from day 1 to 21 dpi, reinforcing the occurrence of prolonged inflammatory responses in these models.^3^ In contrast, we demonstrate that CHIKV- or MAYV-infected mice treated with silymarin showed reduced paw edema compared to infected-only animals, along with less swelling during the peak edema phase.

We also evaluated the dissemination of MAYV and CHIKV in the liver, spleen, quadriceps, paw, soleus, tibialis anterior, and EDL muscles of mice at 7 and 12 dpi. In our study, the treatment with silymarin demonstrated the ability to reduce CHIKV viral load in the spleen and paw pad at 7 dpi and in the soleus, liver, and spleen at 12 dpi. For MAYV, silymarin demonstrated significant antiviral activity, resulting in reduced viral loads in the liver, spleen, quadriceps, and soleus at 7 dpi and in the paw and spleen at 12 dpi. Furthermore, CHIKV was not detected in the quadriceps, tibialis anterior, or EDL muscles in any of the groups, and no statistically significant differences were observed in MAYV and MAYV + SIL viral loads in the EDL and tibialis anterior muscles. The absence of detectable CHIKV RNA in muscles such as the quadriceps, tibialis anterior, and EDL contrasts with the broader tissue distribution observed for MAYV and may reflect fundamental differences in viral tropism, replication kinetics, and host–tissue interactions. Moreover, these temporal and anatomical differences observed in the antiviral activity of silymarin may reflect variations in its pharmacokinetics or tissue-specific bioavailability.

In addition to its anti-inflammatory properties, silymarin is also notable for its broad-spectrum antiviral activity, covering a wide range of viruses including several arboviruses. Silymarin demonstrated antiviral efficacy against hepatitis C virus (HCV),^14^ human immunodeficiency virus type 1 (HIV-1),^15^ and influenza A virus (IAV).^16^ In the context of arboviral infections, silymarin has exhibited antiviral activity against CHIKV *in vitro*,^9^ as well as against MAYV^2^ and ZIKV.^17^ Specifically, for CHIKV, silymarin significantly reduced viral replication efficiency and the production of viral proteins involved in the replication process *in vitro*.^9^ Beyond *in vitro* observations, *in vivo* studies have demonstrated that systemic administration of silymarin or its major flavonolignans exerts pharmacological effects in viral infection models, in which immunomodulatory and antioxidant properties were associated with reduced viral burden and attenuation of tissue damage, particularly in target organs such as liver and lung, in models of MAYV, ZIKV, and IAV infection.^8,18,19^ Importantly, the interpretation of these antiviral effects requires a pharmacokinetic context. Flavonolignans from silymarin are known to display low oral bioavailability due to rapid metabolism and biliary excretion; however, despite limited plasma levels, preferential accumulation in the liver and other tissues has been reported *in vivo*. Moreover, formulation strategies such as phospholipid complexes, self-microemulsifying drug delivery systems (SMEDDS), nanoparticle-based carriers, and intravenous preparations have been shown to substantially increase systemic and hepatic exposure, improving absorption and maximal blood concentrations.^20,21^

CHIKV and MAYV infections also demonstrated significant hepatic damage, including hemorrhages, necrosis, and inflammatory foci within the hepatic parenchyma. However, silymarin treatment effectively reversed or attenuated these damages. In CHIKV-infected animals treated with silymarin, an improvement in the hepatic inflammatory status was observed, characterized by a reduction in inflammatory cells and the presence of smaller and less numerous degenerative areas. Similarly, MAYV-infected animals that were treated also showed a reduction in the hepatic inflammatory infiltrate. Although, the presence of hyperemia was observed in the treated groups, this finding may be related to the possible vasodilatory action of silymarin, as described by Pourová et al.^22^ These results reinforce the previously described effects of silymarin, which include hepatoprotective, anti-inflammatory and antioxidant properties, as demonstrated in our previous study with BALB/c mice infected with MAYV.^8^ Furthermore, silymarin was able to reduce the number and total area of lymphoid nodules at 12 dpi, indicating a possible modulation of the systemic inflammatory immune response.

Literature data indicate that the severity of arthritis and myositis symptoms correlates with cytokine expression profiles during CHIKV and MAYV infections.^6,23–27^ In our present study, histopathological and morphometric analyses demonstrated a significant increase in the number of inflammatory cells in the quadriceps, soleus, tibialis anterior, and EDL in both CHIKV- and MAYV-infected animals, indicating myositis induced by these viruses. Additionally, an increase in the level of inflammatory infiltrate in the paw muscles, bone marrow, and joints was observed in all infected animals. This histopathological triad suggests a comprehensive picture of myositis, arthritis, and bone marrow inflammation in the paws of CHIKV- and MAYV-infected animals. Furthermore, we observed a significant increase in TNF levels in the quadriceps and paws of CHIKV- or MAYV-infected animals. Increased TNF production is recognized as a common biomarker in both chronic CHIKV-associated arthritis and rheumatoid arthritis.^12,25,26^ Moreover, it was evidenced that increased TNF levels significantly contributed to joint damage and hypernociception induced by CHIKV infection.^25,28^ In the case of MAYV, inflammatory mediators such as TNF, IL-6, IFN-γ, and MCP-1 (monocyte chemoattractant protein-1) exhibited elevated concentrations in the serum of infected animals, suggesting an important role in the pathogenesis of the infection.^4^ Notably, in RAG–/– knockout mice (mice lacking the immunoglobulin recombination activating gene and T-cell receptors) infected with MAYV, no tissue damage was found, even after a prolonged period of active MAYV replication. This is very likely due to the absence of TNF, IL-6, and other inflammatory cytokines, which are typically induced as a consequence of lymphocyte-mediated responses. In addition, MAYV replication in macrophages induces TNF expression, which possibly contributes to MAYV pathogenesis by promoting an inflammatory profile characteristic of arthritis, similar to what occurs with CHIKV.^12,29^ In this regard, da Silva et al. demonstrated that in mice infected with CHIKV and MAYV, inflammatory mediators were involved in muscle atrophy, and that TNF neutralization and oxidative stress reduction led to decreased expression of atrogenes and a reduction in atrophic muscle fibers.^24^ Thus, inflammatory mediators produced by immune cells, such as TNF, can be considered critical factors in the development of CHIKV- and MAYV-induced arthritis and myositis.

The administration of silymarin demonstrated a significant anti-inflammatory effect, evidenced by a reduction in the number of inflammatory cells in infected and subsequently treated muscles, which resulted in improved myositis in the quadriceps, tibialis anterior, soleus, EDL, and paw muscles. This benefit was consistently observed in all muscles infected by MAYV. For muscles infected by CHIKV, a reduction in the inflammatory infiltrate was noted in all muscles, with the exception of the soleus, only at 7 dpi. Beyond myositis, silymarin also showed the ability to reduce inflammation in joints and adjacent tissues, promoting a remarkable improvement in arthritis. Concurrently, an attenuation of inflammation in the bone marrow and paws of the animals was observed. Additionally, the anti-inflammatory action of silymarin was confirmed by the reduced expression of TNF in the paws of CHIKV- and MAYV-infected animals at 7 and 12 dpi. This decrease in TNF expression was also observed in the quadriceps muscle for both viruses at 12 dpi.

Studies show that silymarin exerts anti-inflammatory effects mainly through the inhibition of NF-κB activation via the blockage of IκB protein degradation. So, NF-κB is prevented from translocating to the nucleus and interacting with specific DNA regions responsible for the activation of genes involved in the inflammatory response. Consequently, the expression of proinflammatory cytokines, including IL-1β and TNF, can be reduced.^11,30^ This mechanism is consistent with experimental evidence showing a reduced level of cytokine production following silymarin or silibinin treatment. *In vivo*, silibinin significantly decreased levels of IL-1α, IL-6, IL-9, IL-13, IL-16, IFN-γ, and TNF in A/J mice with lung adenocarcinomas.^31^ Similarly, in vitro exposure to silymarin (100 μM) markedly reduced IL-2, IL-10, IFN-γ, TNF, and G-CSF production by activated T lymphocytes after 72 h compared with controls.^32^

These findings highlight the potential of anti-inflammatory strategies for the treatment of arthritis and myositis induced by CHIKV and MAYV. Cytokines and chemokines play critical roles in other viral arthropathies, such as epidemic polyarthritis (EPA) caused by Ross River virus (RRV), as well as in autoimmune conditions such as rheumatoid arthritis (RA). Therefore, the immunopathogenesis of chronic arthritis caused by CHIKV and MAYV may share similarities with these conditions.^12,23,29^ In this context, silymarin has demonstrated the ability to promote an anti-inflammatory profile, likely through the downregulation of proinflammatory cytokine activity. This action may significantly contribute to mitigating disease severity associated with elevated levels of these inflammatory mediators.

## Conclusion

The data obtained in this study highlighted silymarin as a promising therapeutic candidate, exhibiting broad antiviral and anti-inflammatory activities in chikungunya and Mayaro fever. Its administration in CHIKV- or MAYV-infected BALB/c mice led to a reduction in the number and area of lymphoid nodules, a significant reduction in inflammatory infiltrates in liver, muscle, joints, and bone marrow, as well as a decrease in TNF expression, a cytokine that may play a central role in the clinical manifestations associated with these arboviral infections. The ability of silymarin to modulate the inflammatory response combined with its antiviral activity reinforces its potential not only for symptomatic relief but also for the control of viral replication. Thus, the findings presented here suggest silymarin as a promising candidate for managing CHIKV and MAYV infections, with beneficial effects on arthritis and myositis. However, despite the promising antiviral and anti-inflammatory effects observed, the present study is limited by the use of an acute murine model, the restricted evaluation period, and the absence of pharmacokinetic and tissue-distribution analyses of silymarin, which should be addressed in future studies to better define dose–exposure relationships and translational relevance to CHIKV- and MAYV-human disease and thus validate its application.

## Methods

### Virus and Cells

CHIKV S27 African (GenBank accession number AF369024) was isolated in Africa from a feverish patient and was kindly provided by Professor Eurico de Arruda Neto (Medical University of Ribeirão Preto – USP, Brazil). MAYV strain BeAr20290 (GenBank accession number KY618127) was originally isolated from a pool of 93 *Haemagogus* spp., captured 87 km from the Belém-Brasília highway in 1960, and was kindly provided by Professor Maurício Lacerda Nogueira (Medical University of São José do Rio Preto—FAMERP, Brazil). CHIKV (passage 5) and MAYV (passage 3) stocks were propagated in Vero cells (ATCC CCL-81) maintained in Dulbecco’s modified Eagle’s medium (DMEM; Sigma-Aldrich, USA) supplemented with 2% fetal bovine serum (FBS; Gibco, USA), 100 U/mL penicillin, 100 μg/mL streptomycin, and 2.5 μg/mL amphotericin B (Sigma-Aldrich, USA). Vero cells were infected at a multiplicity of infection (MOI) of 0.01 and incubated at 37°C and 5% CO_2_. The supernatant was collected 24 h postinfection (hpi) for CHIKV and 48 hpi for MAYV, clarified by centrifugation at 3500 × *g* for 10 min to remove cell debris, aliquoted, and stored at −80°C. The viral titers were determined by Dulbecco’s plate assay (plaque assay) on Vero cells and expressed as plaque-forming units per milliliter (PFU/mL).

### Silymarin

Silymarin was obtained commercially from Sigma-Aldrich (USA). A vehicle solution of water and carboxymethylcellulose (CMC, 0.5% v/v) was used to dilute silymarin so that the animals could be treated via gavage. The animals were treated with 200 mg/kg/day of silymarin, with the dose divided every 12 h, starting 6 h postinfection. The dose of silymarin was based on previous studies carried out by our study group,^8,18^ as well as on pharmacokinetic studies demonstrating that, although the oral absorption of silymarin is low, plasma levels of 500 mg/L were achieved 90 min after oral administration of 200 mg/kg of silymarin in mice. Furthermore, peak plasma concentrations are reached 4 to 6 h after administration in both humans and animals, with an elimination half-life ranging from 6 to 8 h.^33^

In order to estimate the equivalent human dose, the equation proposed by Reagan-Shaw et al. was utilized.^34^ This equation is based on a conversion factor related to body surface area, a parameter that directly influences drug absorption and bioavailability. Applying this methodology, a dose of 200 mg/kg in mice corresponds to approximately 16.2 mg/kg in humans. Considering an individual with an average weight of 60 kg, the equivalent dose would be about 1000 mg per day. This dosage falls within the safety profile of the substance, as oral doses of up to 2.1 g per day have been considered safe and well tolerated in human patients.^35^

### Ethics Statements

Animal experiments were carried out in accordance with the regulations of the Ethics Committee on the Use of Animals (CEUA) of the Federal University of Ouro Preto (UFOP, Brazil), protocol numbers 4528291122 (ID 000886) and 6633140423 (ID 000885). Young male or female wild-type BALB/c mice (6 weeks old) were acquired and maintained by the Animal Science Center (CCA) at UFOP.

### Mouse Infection

Six-week-old male and female BALB/c mice were randomly assigned to five experimental groups (*n* = 8 per group): uninfected control; CHIKV-infected; CHIKV-infected and treated with silymarin; MAYV-infected; and MAYV-infected and treated with silymarin. The sample size for each experimental group (*n* = 8) was determined using BioEstat software (version 5.3) based on variability observed in pilot experiments.

Animals were housed in polypropylene cages with a microisolator system containing wood shavings under controlled environmental conditions, including temperature, ventilation, and a 12 h light/dark cycle. Throughout the experiment, a standard diet and water were provided ad libitum.

Control animals received an injection of culture medium into the right hind paw pad, whereas infected groups were inoculated with 10^6^ plaque-forming units (PFU) of CHIKV or MAYV via the same route. Silymarin-treated animals received 200 mg/kg/day of silymarin orally by gavage every 12 h, starting 6 h postinfection and continuing for 21 days. The vehicle solution (without silymarin) was administered to the control and untreated infected groups.

First, animals were monitored daily for 21 days to assess clinical progression, including signs such as piloerection, prostration, weight loss, hind limb weakness, joint and muscle swelling, and impaired locomotion. Euthanasia was performed using an anesthetic overdose followed by exsanguination via abdominal aorta transection after the confirmation of an adequate anesthetic plane. The animals received oral tramadol hydrochloride (30 mg/kg) administered 30 min prior to anesthesia induced with ketamine hydrochloride (300 mg/kg) and xylazine hydrochloride (30 mg/kg), administered intraperitoneally, following the National Council for the Control of Animal Experimentation (CONCEA) guidelines for the care and use of laboratory animals in compliance with international laws and policies (CEUA-UFOP 4528291122 and 6633140423).

Based on pilot data, two time points were selected for euthanasia and tissue collection: 7 and 12 days postinfection. All other experimental conditions mirrored those of the pilot study including animal handling, anesthesia, and euthanasia procedures. After euthanasia, tissues including liver, spleen, paws, quadriceps, extensor digitorum longus (EDL), tibialis anterior, and soleus muscles were collected for virological and histopathological analyses to evaluate the antiviral and anti-inflammatory effects of silymarin during different stages of alphavirus-induced arthritis and myositis.

### Paw Edema Assessment

Paw edema was measured daily for 21 days using a Starrett digital caliper and recorded in millimeters. For each animal, the edema was determined by averaging three consecutive measurements, applying minimal pressure sufficient only to induce a slight separation of the toes. In order to ensure consistency and reproducibility, all measurements were performed at the same anatomical site, at a fixed time of day, and by the same trained evaluator throughout our study.

### RNA Extraction and cDNA Synthesis

Total RNA was extracted from all collected organs using the SV Total RNA Isolation System kit (no. Z3105, Promega, USA), following the manufacturer’s instructions. This kit includes an on-column DNase I treatment, which was performed to eliminate genomic DNA contamination. RNA was eluted in nuclease-free water and quantified using a NanoDrop spectrophotometer (Thermo Scientific, USA), with concentrations expressed in ng/μL. RNA purity was assessed by the absorbance ratio at 260/280 nm, and only samples presenting ratios between 1.8 and 2.1 were used for downstream analyses. Complementary DNA (cDNA) was then synthesized from 2 μg of total RNA using MultiScribe reverse transcriptase (50 U/μL) and random primers (GeneAmp RNA PCR kit, Applied Biosystems, USA), according to the recommendations of the manufacturer. The reaction mix also included 100 mM dNTPs, 10× RT buffer, and nuclease-free water. Reactions were performed in a Veriti Thermal Cycler (Applied Biosystems, USA) under the following conditions: 25 °C for 10 min, 37 °C for 120 min, and 85 °C for 5 min, in a final volume of 20 μL. Synthesized cDNA was stored at −20 °C until further use.

### Quantification of Viral Load by RT-qPCR

Viral RNA levels of CHIKV and MAYV in all collected tissues were quantified by real-time PCR (qPCR) using an ABI 7500 Real-Time PCR System (Applied Biosystems, USA). Reactions were performed with a SYBR Green PCR Master Mix (Applied Biosystems, USA), following the protocol of the manufacturer. cDNA synthesized in the previous step served as the template, along with specific primers for CHIKV^36^ and MAYV (Forward: 5′-AAGCTCTTCCTCTGCATTGC-3′ and Reverse: 5′-TGCTGGAAATGCTCTTTGTA-3′). Amplification consisted of 40 cycles at 95 °C for 15 s and 60 °C for 1 min. For semiquantitative analysis, standard curves were generated using RNA extracted from viral stocks of known concentrations (PFU/mL) for both CHIKV and MAYV.

### Histological Analysis

Liver, spleen, paws, quadriceps, extensor digitorum longus (EDL), tibialis anterior, and soleus muscle samples were fixed in 3.7% buffered formaldehyde, dehydrated through graded ethanol, and embedded in paraffin. Tissue sections were prepared and stained with hematoxylin and eosin (H&E) for histological analysis. Paws were demineralized by immersion in a 14% (w/v) aqueous solution of ethylenediaminetetraacetic acid (EDTA) for 21 days, with the solution replaced every 48 h. Morphometric measurements of hepatic inflammatory cells, total cells, and the area of white pulp from the spleen (25 sections/animal) were performed using an optical microscope at 400× or 200× magnification (Leica DM5000B). Liver, paws, and muscle analyses were performed using Leica QwinV3 Image Processing and Analysis software (Germany), whereas spleen analyses were performed using ImageJ software.

### TNF-α Measurements by RT-qPCR

TNF-α mRNA levels were quantified by real-time qPCR using the SYBR Green PCR Master Mix (Applied Biosystems, USA), following the guidelines of the manufacturer. Total RNA extraction and cDNA synthesis were performed as previously described. GAPDH was used as the endogenous reference gene (normalizer). Primer sequences for TNF-α were obtained from Figueiredo et al.^37^ and for GAPDH from Xiong et al.^38^

### Statistical Analysis

Viral load data and relative quantification of TNF-α expression were analyzed using Student’s *t* test. Morphometric analyses were performed using one-way ANOVA followed by Tukey’s multiple comparison test, whereas paw pad measurements were analyzed using two-way ANOVA. Correlation analyses between TNF-α levels and morphometric parameters of tissue inflammation were performed using Pearson’s or Spearman’s correlation coefficients (Prism 9, GraphPad Software, Inc., USA). Data normality was assessed using the Shapiro–Wilk test. Results are expressed as the mean ± standard deviation (SD), and *p* values <0.05 were considered statistically significant. The symbols *, **, ***, and **** represent significant differences between the groups with *p* ≤ 0.05, *p* ≤ 0.01, *p* ≤ 0.001, and *p* ≤ 0.0001, respectively. Standard curves for viral load quantification by RT-qPCR were generated by linear regression analysis using Microsoft Excel.

## Supplementary Material







## Notes

Animal Ethics Statements for Chikungunya virus (PDF)
Animal Ethics Statements for Mayaro virus (PDF)
Table S1: Inflammatory cells in the liver at 7 and 12 DPI (mean ± SD) (PDF)

## Abbreviations

CF: chikungunya fever
CHIKV: chikungunya virus
dpi: day postinfection
EDL: extensor digitorum longus muscle
GAPDH: glyceraldehyde-3-phosphate dehydrogenase
MOI: multiplicity of Infection
PCR: polymerase chain reaction
PFU: plaque forming units
SIL: silymarin
MAYV −: Mayaro virus
MF: Mayaro fever
