# Supplementary material for: Silymarin Attenuates Arthritis and Myositis in a Murine Model of Acute Infection by Chikungunya and Mayaro Viruses
Source: ACS Infect Dis. 2026 Jan 23;12(2):738–49. doi: 10.1021/acsinfecdis.5c00901 (PMC12910590; doi:10.1021/acsinfecdis.5c00901)
Supplement: Supplementary file 1 [file id5c00901_si_001.pdf]

CERTIFICADO

Certificamos que a proposta intitulada "Avaliação da silimarina frente a infecção pelo vírus Chikungunya ", protocolada sob o CEUA nº 4528291122 (ID 000886), sob a responsabilidade de **Cintia Lopes de Brito Magalhães** *e equipe; Rafaela Lameira Souza Lima* - que envolve a produção, manutenção e/ou utilização de animais pertencentes ao filo Chordata, subfilo Vertebrata (exceto o homem), para fins de pesquisa científica ou ensino - está de acordo com os preceitos da Lei 11.794 de 8 de outubro de 2008, com o Decreto 6.899 de 15 de julho de 2009, bem como com as normas editadas pelo Conselho Nacional de Controle da Experimentação Animal (CONCEA), e foi **APROVADA** pela Comissão de Ética no Uso de Animais da Universidade Federal de Ouro Preto (CEUA/UFOP) na reunião de 24/05/2023.

We certify that the proposal "Evaluation of silymarin against Chikungunya virus infection ", utilizing 168 Isogenics mice (males and females), protocol number CEUA 4528291122 (ID 000886), under the responsibility of **Cintia Lopes de Brito Magalhães** *and team; Rafaela Lameira Souza Lima* - which involves the production, maintenance and/or use of animals belonging to the phylum Chordata, subphylum Vertebrata (except human beings), for scientific research purposes or teaching - is in accordance with Law 11.794 of October 8, 2008, Decree 6899 of July 15, 2009, as well as with the rules issued by the National Council for Control of Animal Experimentation (CONCEA), and was **APPROVED** by the Ethic Committee on Animal Use of the Ouro Preto Federal University (CEUA/UFOP) in the meeting of 05/24/2023.

Finalidade da Proposta: Pesquisa (Acadêmica)

Vigência da Proposta: de 04/2023 a 12/2024 Área: Ciências Biológicas

Origem: Centro de Ciência Animal
Espécie: Camundongos isogênicos sexo: Machos e Fêmeas idade: 15 a 42 semanas Quantidade: 168
Linhagem: Balb/c Peso: 6 a 35 g

Ouro Preto, 25 de maio de 2023

Wanderson G. de Lima

Prof. Dr. Wanderson Geraldo de Lima
Coordenador da Comissão de Ética no Uso de Animais
Universidade Federal de Ouro Preto

Profa. Dra. Cintia Lopes de Brito Magalhães
Vice-Coordenador da Comissão de Ética no Uso de Animais
Universidade Federal de Ouro Preto
