# Supplementary material for: Silymarin Attenuates Arthritis and Myositis in a Murine Model of Acute Infection by Chikungunya and Mayaro Viruses
Source: ACS Infect Dis. 2026 Jan 23;12(2):738–49. doi: 10.1021/acsinfecdis.5c00901 (PMC12910590; doi:10.1021/acsinfecdis.5c00901)
Supplement: Supplementary file 3 [file id5c00901_si_003.pdf]

Table S1. Inflammatory cells in the liver at 7 and 12 DPI (mean ± SD)

Group	n	7 DPI (mean ± SD)	12 DPI (mean ± SD)
Control	5	14.40 ± 1.39	14.50 ± 2,13
CHIKV	5	18.76 ± 2.51	20.09 ± 4,77
CHIKV+ SIL	5	14.02 ± 0.97	14.40 ± 0,91
MAYV	5	35.46 ± 9,95	35.10 ± 5,16
MAYV + SIL	5	19.81 ± 3,22	21.20 ± 1,75

Table S1. **Inflammatory cells in the liver at 7 and 12 DPI**. Morphometric analysis of liver inflammatory cell counts showing reduced inflammatory infiltrates in CHIKV- and MAYV-infected mice treated with silymarin. Data are expressed as mean ± SD. Statistical analyses are presented in the corresponding figures (Figure 4).
